# Supplementary material for: Relationship between Strength and Professional Quality of Life of Nurses Working Hospital Emergency Departments
Source: Int J Environ Res Public Health. 2023 Jan 22;20(3):2052. doi: 10.3390/ijerph20032052 (PMC9914948; doi:10.3390/ijerph20032052)
Supplement: Supplementary file 1 [file ijerph-20-02052-s001.zip › ijerph-2115244-SI.pdf]

Questions of CVP-35

1. Amount of work I have
2. Satisfaction with the type of work
3. Satisfaction with salary
4. Possibility of promotion
5. Recognition of my effort
6. Pressure I receive to perform the amount of work
7. Pressure received to maintain the quality of my work
8. Rush and stress due to lack of time to do my work
9. Motivation (desire to make an effort)
10. Support from my bosses
11. Support from my colleagues
12. Support from my family
13. Desire to be creative
14. Possibility to be creative
15. Disconnect at the end of the workday
16. I receive information on the results of my work
17. Conflicts with other people at work
18. Lack of time for my personal life
19. Physical discomfort at work
20. Ability to express what I think and need
21. Disclaimer
22. My company tries to improve the quality of life of my position
23. I have autonomy or freedom of decision
24. Nuisance interruptions
25. Stress (emotional exertion)
26. Training needed to do my job
27. I am able to do my current job
28. Variety in my work
29. My work is important to other people's lives
30. It is possible that my proposals will be heard and implemented
31. What I have to do is clear
32. I am proud of my work
33. My work has negative consequences for my health
34. Quality of life of my work
35. Peer support in management responsibilities
